# Self-control Mediates the Relationship between Psychosocial Strengths and Perceived Severity of COVID-19 among Frontline Healthcare Professionals of Pakistan: A Single Center Experience

**DOI:** 10.12669/pjms.36.COVID19-S4.2662

**Published:** 2020-05

**Authors:** Muhammad Saleem, Saima Dastgeer, Areeha Khan Durrani, Abubakr Ali Saad, Zubair Manzoor, Hafiz Nauman Hussain

**Affiliations:** 1 Dr. Muhammad Saleem Associate Professor, Department of Applied Psychology, The Islamia University of Bahawalpur, Pakistan; 2 Saima Dastgeer Assistant Professor (Psychology), Govt. College of Home Economics, Multan, Pakistan; 3 Areeha Khan Durrani, PhD Scholar Department of Applied Psychology, The Islamia University of Bahawalpur, Pakistan; 4 Dr. Abubakr Ali Saad Associate Professor (Cardiology), Dera Ghazi Khan Medical College and Teaching Hospital, Pakistan; 5 Zubair Manzoor - PhD Scholar Department of Applied Psychology, The Islamia University of Bahawalpur, Pakistan; 6 Dr. Hafiz Nauman Hussain Postgraduate Resident (Anesthesia Department) Dera Ghazi Khan Medical College, Pakistan

**Keywords:** COVID-19, psychosocial strengths, perceived severity, self-control, frontline healthcare professionals

## Abstract

**Objective:**

To examine the relationship between psychosocial strengths (resilience, self-efficacy beliefs and social support) and perceived severity of COVID-19 and also to gauge the mediating role of self-control among frontline health care professionals of Pakistan.

**Methods:**

A cross-sectional research design was utilized from March to April 2020 from one medical teaching hospital of South Punjab. As it was a single center experience so all the doctors were approached and asked to participate in this research. In total, 284 doctors (out of 300 approx.) completed online survey. The data were collected through online google forms consisting of self-report measures i.e. Brief Resilience Scale, Short General Self Efficacy Scale, Brief Scale for Social Support, Risk Behavior Diagnostic Scale and Brief Self-Control Scale.

**Results:**

The results were analyzed by using SmartPLS (3.0), direct effect of psychosocial strengths on perceived severity of COVID-19 and indirect effect of self-control were assessed through path coefficients, t-values and r-square values. The results confirmed that there was significant negative relationship between psychosocial strengths and perceived severity of COVID-19 (β = -0.854, t =14.279) with 72% variance in perceived severity due to psychosocial strengths. Further, the results also suggest that self-control proved significant mediator between psychosocial strengths and perceived severity (β = -0.604, t = 11.004, variance in perceived severity is 74%).

**Conclusion:**

In the time of pandemic, medical professionals are working as frontline force and can have several uncertainties regarding the risk associated with outbreak of COVID-19. This study concludes psychosocial strengths can play a significant role in subsiding the risk associated with severity of disease. Whereas, self-control can significantly contribute to buffer the negative influence of COVID-19 among frontline medical professionals. In line with findings of this study, there is a dire need to initiate psychotherapeutic studies for medical professionals to boost up their psychosocial strengths that would make them resilient against COVID-19.

## INTRODUCTION

The outbreak of COVID-19 has created a sense of rapid emergency, everyone is victim of varied psychological responses.^1^ It is not only an extraordinary public health concern but also a huge distressing factor for the healthcare providers and medical staff too.^2^ Health professionals, especially those working in hospitals caring for people with confirmed or suspected COVID-19 cases, are more vulnerable to become victim of this deadly virus. That’s why they are more prone to develop psychosocial health issues. In the same vein, a study conducted in Beijing, at the time of SARS outbreak exhibits that fear of contagion, depression, anxiety, and frustration is reported alarmingly high among frontline healthcare professionals; especially who are dealing with SARS-COV-2 positive patients.^3^-^4^ Similarly, a recent study at Wuhan also reported that medical staff who had previous contact with COVID-19 patients exhibited high levels of psychosocial problems including fear of contagion.^5^

It is reasonable to assume that outrageous growth is evident in the number of positive COVID-19 patients, why we should not quantify the role of protective shields (psychosocial strengths) that directly or indirectly safeguard psychological health of frontline medical professionals. These psychosocial strengths are key elements that boost well-being of individuals facing adversity.^6^,^7^ Further, it has been reported that self-control is prime factor for the cultivation of resilience and other psychosocial strengths among individuals.^8^ In general, self-efficacy is also an important factor that influences an individual’s ability to exert self-control.^9^,^10^ It is established that resilience, efficacy beliefs, accepting challenges and positive coping strategies reduce perceived severity of disease in individuals. 11 Prior studies on pandemics, found that good self-control is not directly link to psychosocial strengths but also intimidate negative effects, particularly, impact of perceived severity of disease. 12-14

In the light of above cited scenario, current study sought to examine the relationship between psychosocial strengths and perceived severity of COVID-19 through the lens of self-control among frontline healthcare professionals of Pakistan.

## METHODS

The cross-sectional research was conducted in a teaching and medical hospital of South Punjab, where medical professionals are directly exposed to dealing with COVID-19 patients. It was a single center research so all frontline healthcare professionals (i.e. doctors) were approached to participate in this study. Study conducted from March to April, 2020. There were total 300 doctors deputed as frontline professionals and 284 willingly filled the online survey (through google forms) consisting of self-administrated questionnaires

Psychosocial strengths were measured through three scales i.e. Brief Resilience Scale (BRS)^15^ a six items short scale to measure resilience, Short General Self-Efficacy Scale (SGSE)^16^ is also a six items inventory and measures self-efficacy beliefs within an individual and Brief Scale for Social Support (BSSC) consist of nine items which measure the emotional, interpersonal and material support.^17^ Further, Risk Behavior Diagnostic (RBD) scale is three items measures which was used to measure perceived severity of COVID-19^18^ and Brief Self-Control Scale (BSCS) was used to measure self-control.^19^

The ethical approval was obtained from ethical research committee of concerned department institution. The informed consent was also acquired from the participants and they were also ensured about the confidentiality of information they provided. Additionally, permission to employ measurement instruments was also obtained from the authors/institutions, wherever desired.

The data were analyzed through SmartPLS (3.0). To check psychometric estimates of measurement instruments, reliability and validity tests were employed. Further, the effect of psychosocial strengths on perceived severity (direct effects of three IVs) and mediating role of self-control (indirect effect of mediator) were observed through path coefficients (β), r-square and effect size through f^2^. Whereas, the t-values were considered for the significance of model through bootstrapping and Q^2^ values depicted the relevance of model through blindfolding.

## RESULTS

**Fig.1:**
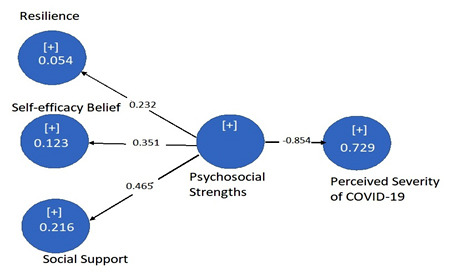
This figure demonstrates the path coefficients and variance in perceived severity of COVID-19 due to psychosocial strengths.

**Fig.2:**
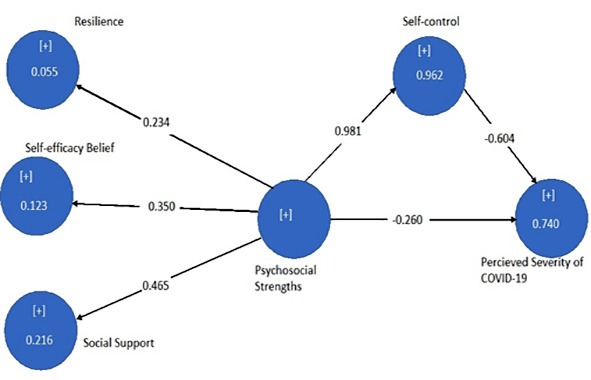
This figure shows the mediating role of self-control with path coefficient and variance in the perceived severity due to mediating variable.

The baseline characteristics of respondents were obtained through the frequency distribution of gender; among 284 respondents, 176 were male (62%) and 108 were females (38%). Cronbach’s alpha, composite reliability, average variance estimated and discriminant validity are shown in Table-I. The psychosocial strengths (resilience, self-efficacy beliefs and social support) were significantly negatively corelated with perceived severity of COVID-19 and the values for path coefficient can be seen in Table-II and Fig.1. Whereas, the mediation model shows significant mediating role of self-control in Table-III and Fig.2.

**Table-I:** Reliability and Validity estimates of constructs.

Variables	Cronbach’s Alpha	Composite Reliability	AVE	Discriminant Validity
BRS	0.86	0.90	0.602	Yes
SGSE	0.93	0.95	0.761	Yes
BSSC	0.82	0.91	0.651	Yes
RBDS	0.89	0.93	0.624	Yes
BSCS	0.81	0.88	0.655	Yes

Note: BRS= Brief Resilience Scale; SGSE= Short General Self-Efficacy Scale;

BSSC= Brief Scale for Social Support; RBDS=Risk Behavior Diagnostics Scale;

BSCS= Brief Self-control Scale

**Table-II:** Direct effect of Psychosocial Strengths on Perceived Severity of COVID-19 among Frontline Healthcare Professionals of Pakistan (N=284).

Relationship	Path coefficient	Mean	SD	t-value	p value	R^2^	Ad. R^2^	f^2^	Q^2^
PSS >PS	-0.854	0.728	0.061	14.279***	0.000	0.729	0.73	0.221	0.444

Note: PSS= Psychosocial Strengths; PS= Perceived Severity;

*** Significance at 1%

**Table-III:** Indirect effect of Psychosocial Strengths on Perceived Severity of COVID-19 among Frontline Healthcare Professionals of Pakistan through Self-control (mediator) (N=284)

Relationship	Path coefficient	Mean	SD		t-value	p value	R^2^	Ad. R^2^	f^2^	Q^2^
PSS>PS	-0.260	0.442	0.135	3.123***		0.002	-	-	-	-
PSS>SC	0.981	0.920	0.019	49.134***		0.000	-	-	-	-
SC>PS	-0.604	1.265	0.113	11.004***		0.000	-	-	-	-
PSS>SC>PS	-	-	-	-		-	0.74	0.72	2.690	0.402

Note: PSS= Psychosocial Strengths; PS= Perceived Severity;

SC=Self-control;

*** Significance at 1%

The reliability and validity estimates of the constructs are given in Table-I.. For psychosocial strengths, three constructs for resilience, self-efficacy and social support measures were used. In the table, Average Variance Estimated (AVE) for all scales are acceptable when composite reliability is above than 0.60 according to Fornell-Larcker Criterion method.^20^ Moreover, the composite reliability (> 0.80) and Cronbach Alpha (> 0.80) for all scales demonstrates good internal consistency reliability of these measures.

The direct effect of psychosocial strengths on perceived severity of COVID-19 on the frontline health care professionals with the significant negative correlation, (path coefficient), mean, SD, t value, p value, R square, effect size and cross validation redundancy estimates for model relevancy is shown in Table-II.

The mediating role of self-control between psychosocial strengths and perceived severity of COVID-19 among the frontline health care professionals with the correlation value (path coefficient), mean, SD, t value, p value, R square, effect size and cross validated redundancy estimates for relevancy of model is shown in Table-III. Here it can be clearly seen that self-control is a strong mediator between psychosocial strengths and perceived severity of disease.

## DISCUSSION

This study proved significant negative correlation between psychosocial strengths and perceived severity of COVID-19 among health care professionals. It clearly depicts the importance of psychosocial strengths that safeguard medical professionals if these shields are well-nurtured.^21^ Although, some of the psychosocial strengths are in-built, man cannot polish or boost them, but few are learned and can be refined with psychological training. At the time of crisis, we can select our medical professionals with high level of these strengths, so that other cohorts can be protected.

Plenty of studies proved that resilience and perceived social support can minimize the perceived severity of disease.^6^,^11^,^12^ As Pakistani we are used to live in collectivistic settings either in homes or outside. This naturally provides social support all the time. At the time of adversity, this need is multiplied. It is good and prudent finding for those medical professionals whom facing this hardship directly, must maintain their social contact with others – family, friends, and significant others. Although it is advised to maintain a social distance to remain safe from SARS-CoV-2. In real terms, it is necessary to maintain physical distance rather social distance; here is a dire need to stay connected (either utilizing remote means) with others to get social support to maintain good mental health. Similarly, World Health Organization also suggesting the frontline medical professionals to take care their stress level and mental health along with physical health.^22^

Self-control is also seen as mediating factor between psychosocial strengths and severity of COVID-19, that remined significant with 74% variance. It shows that we cannot minimize the role of self-control, when enhancing the psychosocial strengths of medical professionals. Self-control buffer in reducing the negative influence of perceived severity.^12^,^13^

The current findings illustrate that prevalence of psychosocial strengths i.e. resilience, self-efficacy beliefs and social support in the health care providers, facilitate to manage the risk of pandemic related stigma and fears. Further, self-control is the strong psychological strategy for enhancing all the positive coping mechanisms within the frontline doctors. In Pakistan, this is the first study that encompasses psychosocial needs of frontline medical heroes that protect their mental health if well-cherished.

### Limitations and future avenues of study

Considering as a baseline research, this study collected data from doctors only, it is expected to include allied medical staff in upcoming researches. It was a single center experience, so we suggest the veterans to extend this study on other quarantine centers of Pakistan. There are several other psychosocial factors which need to be addressed and qualitative research should also be commissioned to understand the in-depth and lived experiences of affected.

## CONCLUSIONS

Psychosocial strengths are very important indicators for minimizing the risk related to pandemic COVID-19. At this time of crisis, several stressors are faced by the health care professionals but those who have strong self-control, have resilient personality, self-efficacy beliefs and adequate social support can cope up well with the adversity.

### Authors’ Contribution

**MS:** Conceived, designed & did editing of manuscript.

**SD:** Did data collection.

**AKD:** Did statistical analysis and manuscript writing.

**AAS & ZM:** Did literature review.

**HNH:** Did review and final approval of manuscript.
